# Surgical tele-mentoring using a robotic platform: initial experience in a military institution

**DOI:** 10.1007/s00464-023-10484-1

**Published:** 2023-10-11

**Authors:** Hunter Faris, Cyril Harfouche, Jesse Bandle, Gordon Wisbach

**Affiliations:** 1 Department of General Surgery, Navy Medicine Readiness & Training Command –San Diego, 34800 Bob Wilson Drive, San Diego, CA 92134 USA; 2 Virtual Medical Center, Navy Medicine Readiness & Training Command – San Diego, San Diego, CA USA

**Keywords:** Surgical tele-mentoring, Teleproctoring, Robotic surgery, da Vinci system, Remote mentor, Surgery

## Abstract

**Background:**

Surgical tele-mentoring leverages technology by projecting surgical expertise to improve access to care and patient outcomes. We postulate that tele-mentoring will improve surgeon satisfaction, procedural competence, the timeliness of operative intervention, surgical procedure efficiency, and key intra-operative decision-making. As a first step, we performed a pilot study utilizing a proof-of-concept tele-mentoring process during robotic-assisted surgery to determine the effects on the perceptions of all members of the surgical team.

**Methods:**

An IRB-approved prospective feasibility study to determine the safety and efficacy of remote surgical consultation to local surgeons utilizing robotic surgery technology in the fields of general, urology, gynecology and thoracic surgery was performed. Surgical teams were provided a pre-operative face-to-face orientation. During the operation, the mentoring surgeon was located at the same institution in a separate tele-mentoring room. An evaluation was completed pre- and post-operatively by the operative team members and mentor.

**Results:**

Fifteen operative cases were enrolled including seven general surgery, four urology, one gynecology and three thoracic surgery operations. Surveys were collected from 67 paired survey respondents and 15 non-paired mentor respondents. Participation in the operation had a positive effect on participant responses regarding all questions surveyed (*p* < 0.05) indicating value to tele-mentoring integration. Connectivity remained uninterrupted with clear delivery of audio and visual components and no perceived latency. Participant perception of leadership/administrative support was varied.

**Conclusions:**

Surgical tele-mentoring is safe and efficacious in providing remote surgical consultation to local surgeons utilizing robotic surgery technology in a military institution. Operative teams overwhelmingly perceived this capability as beneficial with reliable audio-visual connectivity demonstrated between the main operative room and the Virtual Medical Center. Further study is needed to develop surgical tele-mentoring to improve patient care without geographic limitations during times of peace, war and pandemic outbreaks.

Tele-medicine and the remote gathering of information have seen a revolution with the reality of a global pandemic in 2020. The large scale of the pandemic caused a major shift in delivery of care across health care systems. New technology that facilitates synchronous (real-time), asynchronous (store and forward), and remote monitoring is being integrated and applied across the world leading telehealth to become the fastest growing sector of health care [1]. This growth has seen limitations though primarily when applied to the sector of surgery. There are promising remote surgical technological options available but currently there is limited adoption of the technology inhibiting standardization and commercialization for consistent use [2].

Since the advent of minimally invasive surgery, remote surgical application has been discussed and tested. The facets practiced have grown from remote live surgical demonstrations to remote surgical mentoring to full surgical procedures performed remotely [3]. Unfortunately, current experience is based primarily on animal experiments, simulation models, and a select few cases of remote laparoscopic surgery for patients [2, 4]. Barriers to commercial use of remote surgical technology include patient safety concerns, legal liability, surgical credentialing, economic reimbursement for services, a standardized platform for adoption and integration, and connectivity and latency between components [2, 3, 5–8]. Some of these factors pose less of a barrier in the Military Health System (MHS), as it is committed to expanding telehealth services by advancing technology through sustained collaboration. Telehealth services contribute to the MHS goal of increasing readiness, access, quality, and patient safety to the service member. Though finding surgical technology that can achieve these goals has been challenging.

An application termed ‘tele-mentoring’ that involves remote surgical mentoring transmitted via audio and/or video data has been explored to improve remote surgical applications. The technology has the power to transmit specialized surgical sub-specialists remotely to locations geographically distant and/or hazardous [9, 10]. Tele-mentoring has shown similar results achieved when compared to direct in-room mentoring, documenting its reliability and feasibility [2, 11, 12]. Tele-mentoring technology has been broadly investigated through various methods that so far have failed to identify an ideal platform [13]. Robotic-assisted surgery has fewer tested options but does benefit from the advantage of an almost exclusive platform with the da Vinci Surgical System created by Intuitive Surgical Inc. (Sunnyvale, CA, USA) [14]. This system has some of the best visualization capabilities of any laparoscopic technology used today and already utilizes a mentoring console format that facilitates audio and video transfer across fiber optic cables with no perceived latency.

Robotics was applied to surgery over half a century ago as a military project aimed at replacing the surgeon’s physical presence giving soldiers surgical care in battlefield environments [15]. Many advancements have been achieved where robotic systems have successfully completed tele-surgical operations and even established telerobotic remote surgical services for rural communities [4, 16]. Telehealth application of robotic-assisted platforms has tremendous potential however no platform today is engineered to complete remote surgical procedures. To overcome the obstacles faced in remote application of surgery a sustainable collaboration between key stakeholders like medical professionals, innovative providers and industry needs to identify a platform that will undergo development and rigorous testing to achieve a sustainable means of tele-enable surgical options. Towards this end, we begin the development of the first tele-enabled da Vinci Robotic Surgical System (Intuitive Surgical Inc., Sunnyvale, CA). The da Vinci system has been used in previous tele-surgery enabled platforms to complete pyeloplasty [17] and nephrectomies [18] in animal models with the primary goal of evaluating the connectivity and feasibility of these systems. In addition, the da Vinci Xi system had been used in tele-mentoring endeavors in human models with one study testing over a VPN network during robotic-assisted radical prostatectomies [19], while another used Connect™, a second-generation tele-mentoring interface developed by Intuitive Surgical Inc, during robot-assisted prostatectomies and nephrectomies [11].

Our pilot study aims to evaluate the safety and efficacy of a remote mentoring surgeon, using Ethernet connection via a secure network communicating with the local operating surgeon. This prospective feasibility study demonstrates a ‘proof of concept’ that evaluates the quality of the network and determines the effects on performance for all members of the operating team.

## Materials and methods

### Study design

After institutional review board approval at the Navy Medicine Readiness & Training Command—San Diego (NMCSD.2019.0061), we performed a prospective feasibility study in 2021 of surgical tele-mentoring during robotic-assisted procedures in the specialties of general surgery, urology, gynecology and thoracic surgery. All procedures were standard of care and all instruments/devices in the study were FDA approved. A credentialed and capable staff surgeon was always present in the local operating room participating in the operation.

After agreement by the staff surgeon for enrollment, the entire operative team was enrolled including staff surgeon, assistant/training surgeon, anesthesia service provider, operative nurse and surgical technician as well as the mentor. The patient was pre-operatively provided a Patient Information Sheet about the opportunity for surgical tele-mentoring during the robotic surgery operation and had the choice to opt-in or opt-out of participation in this study through written consent. Participants of the operative team were provided a preoperative face-to-face orientation by the mentor of the planned tele-mentoring session to establish communication technique and terminology.

### Surgical tele-mentoring technique

**Fig. 1:**
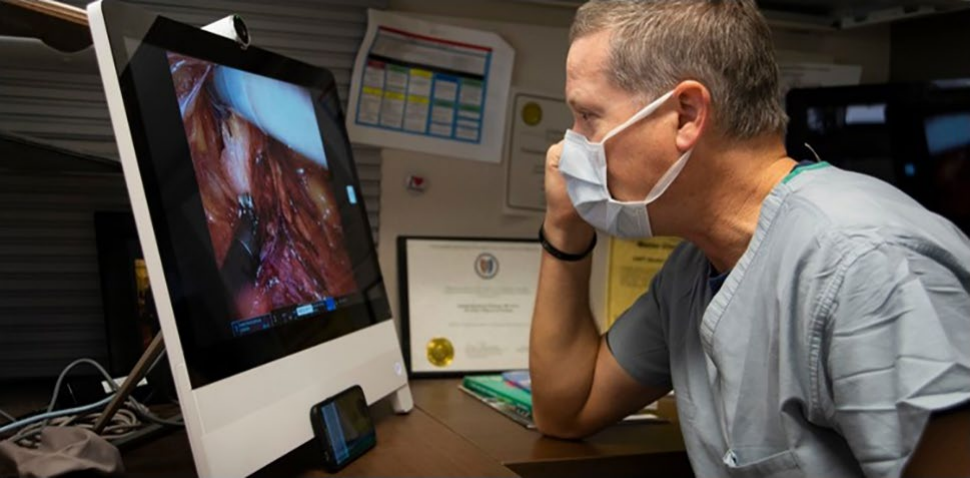
Remote surgical mentor providing oversight and instruction to the local surgeon

The operative team was in either of the two main operating rooms that supported surgery performed with a da Vinci Xi robotic system. These team members focused on delivering safe quality care and were not tasked with setting up tele-mentoring equipment or wearing extra communication devices. The primary and assistant surgeons communicated with the mentor via the dual consoles. The mentor was in the surgical tele-mentoring room in the Virtual Medical Center separate from the operating rooms in an adjacent building approximately 1000 ft apart. The mentor used a two-dimensional Cisco Webex DX80 video teleconferencing (VTC) system device connected via secure network over Ethernet link to communicate with the mentee via Cisco Codec Pro linked with the robotic surgery console (Fig. 1). This link allowed one-way video, two-way audio, and one-way telestration. With these three features, this new remote secure link allowed for surgical technique and anatomy to be communicated and mentored between mentor and surgical team in high fidelity manner. The surgical mentor could provide oversight utilizing any Cisco Webex DX80 VTC system within the military’s secure network.

### Data collection

The data collected included basic operation details, and evaluations of the operative team. Likert Scale-based evaluations were obtained pre- and post-operatively from the operative team members as well as from the mentor post-operatively. Evaluations assessed the team members perception of tele-mentoring to include: satisfaction, effect on level of surgical services offered, patient outcomes, mentor–mentee orientation and relationship, reliability of audio/video connection, leadership and administration support, benefits and challenges to launching tele-mentoring.

### Statistical analysis

Statistical analysis was conducted using Wilcox on matched-pairs signed rank test and sign test of matched pairs to determine statistical significance [20, 21]. The Wilcox on matched-pairs test determined whether the pre- and post-operation survey responses were distributed differently or if there was a definitive change. The sign test of matched pairs provided two forms of information: whether the medians of the two samples are different or not, and whether the post-operational response were numerically higher on average. As each were matched pair tests, individual survey respondent pre- and post-operational responses were paired together providing a more precise analysis. The significance level was designated as 0.05.

## Results

In all, 15 robot-assisted cases were enrolled involving 15 staff surgeons, 10 assistant/trainee surgeons, 15 anesthesia service providers, 15 operative nurses, 15 surgical technicians, and 1 mentor with no cases excluded. Surveys were collected from 67 paired survey respondents and 15 non-paired mentor respondents. Evaluations not received included: one anesthesia pre-evaluation, one pre- and post-evaluation for operative nurses, and one pre and post-evaluation for surgical technicians. 97% of all evaluations were collected. The breakdown of the 15 robotic-assisted cases enrolled was 7 general surgery, 4 urology, 1 gynecology, and 3 thoracic surgery.

**Fig. 2:**
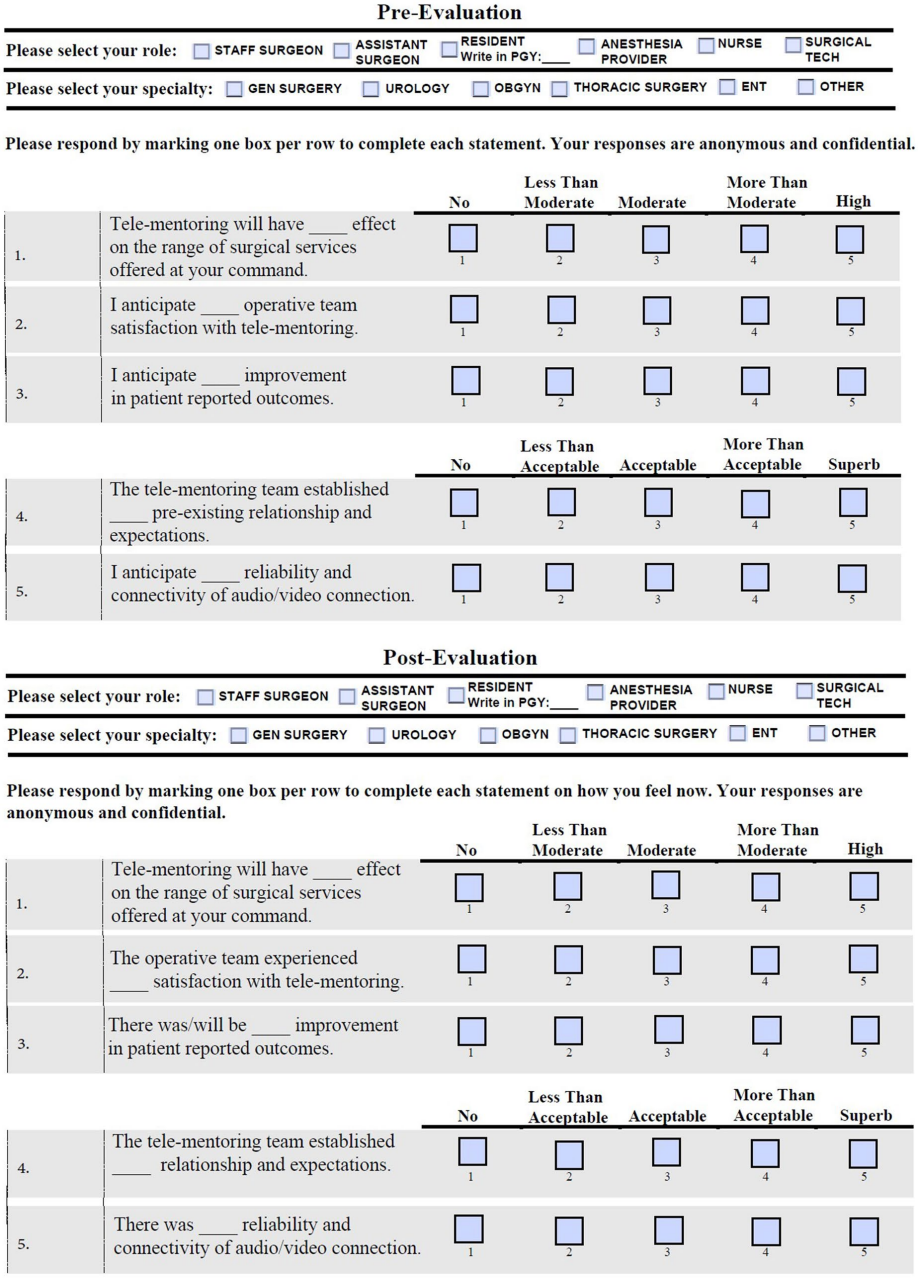
Pre- and post-operative evaluations using a Likert scale

The statistical review indicated that with a strong degree of confidence (*p* < 0.05), participation in the operation had a positive effect on participant response regarding all questions surveyed. After experiencing the integration and use of tele-mentoring, the positive effect median found responses were either “more than moderate” or “more than acceptable” (Table 1). The post-operational question analysis was statistically responded higher by a degree of one on average in the Likert Scale-based evaluations the pre-operational responses (*p* < 0.05) indicating the value of tele-mentoring integration during these surgical cases (Fig. 2). Each statistical test significance was concordant lending extra support to the conclusions outlined (Table 2).

**Table 1:** Pre- and post-operative evaluation results with median response

	Survey questions	Median found in each response
1	Tele-mentoring will have ____ effect on the range of surgical services offered at your command	“a more than moderate” 4
2	The operative team experienced ____ satisfaction with tele-mentoring	“a more than moderate” 4
3	There will be ____ improvement in patient reported outcomes utilizing tele-mentoring	“a more than moderate” 4
4	The tele-mentoring team established ____ relationship and expectations during the case	“a more than acceptable” 4
5	There was ____ reliability and connectivity of audio/video connection during the tele-mentoring case	“a more than acceptable” 4

**Table 2:** Pre- and post-operative evaluation results

Questions	Average response		Wilcoxon test *p*-value	Sign test	
	Pre	Post		Difference of medians *p*-value:	Change > 0:
Q1	3.27	3.51	0.0065	0.0094	0.0047
Q2	3.49	3.89	0.0015	0.0037	0.0019
Q3	3.22	3.53	0.0150	0.0227	0.0113
Q4	3.48	3.80	0.0009	0.0015	0.0008
Q5	3.30	3.72	0.0000	0.0000	0.0000

There were no instances when the connection between teams was lost during the case. Each remote session took place at a mean (range) 1000 ft separation between the surgical team and mentor. The connectivity between robotic console and Cisco Webex DX80 VTC system remained uninterrupted and delivered clear audio and visual components. The mentor had no concerns with the clarity of the video. Mentor and in-room surgical teams’ perception between all cases did not statistically differ between survey evaluations. All interactions were limited to verbal guidance and assistance from mentor to surgical team via the communication platform integrated into the da Vinci Xi system. From survey results, each non-surgeon team member found the tele-mentoring platform to be non-disruptive during the procedure. There were no intra-operative complications reported during cases enrolled. There was no instances in which the da Vinci Xi surgical system couldn’t be used or wasn’t medically indicated.

## Discussion

The integration and adoption of new technologies into the field of surgery has been a long and arduous path. Laparoscopic surgery revolutionized the field but its first uses date back to 1933 when the first laparoscopic lysis of adhesion was performed [22]. Laparoscopic procedures compared to open surgery are now the standard of care in many areas but the slow progress and uptake was related to the technological limitations at the time, and to the requirement of clinical evidence to demonstrate clinical safety and patient benefit [22]. Interestingly robotic surgery is currently going through a similar integration and adoption period with its advocates reporting improved dexterity, better ergonomics, enhanced visualization, decreased fatigue, and less surgeon physiological tremor compared to laparoscopic and open surgery [23]. An additional attribute to the list of robotic surgical benefits is the ability to deliver a dynamic means of teaching, training and oversight. Tele-mentoring is leveraging this technology to provide further growth and utilization. Our present pilot study is a new means of adapting the current da Vinci Xi’s connectivity beyond it’s current capabilities reporting on the clinical safety, feasibility, benefit, and improved surgical experience through this technological advancement.

Safety is a core tenant of providing health care, though recent studies in the USA estimate 1/3 of all medical injuries are due to error [24]. Applications of new technologies like robotic surgery and tele-mentoring have the potential to increase the risk of errors. Currently it is estimated that the annual cost of medical errors to US healthcare is €17.1 billion and 6 of the top 10 identified medical errors are related to surgical procedures [25]. Robotic surgery has been identified in a health technology hazard independent review as one of the top ten risks to US patients due to the lack of robotic surgical training. “Insufficient training of surgeons on robotic technologies can result in surgical errors that lead to prolong surgery, substandard operation outcomes early in the surgeons’ learning curve, complications that require additional treatment and even serious patient injury or death.” stated in an ECRI report [26]. The first validated robotic training curriculum was published in 2015 but there are limits with its application because local experts are limited and may not be available to provide the necessary training to reach curriculum benchmarks [27, 28]. Traditional training and mentoring typically involves a surgical mentor providing guidance via direct verbal communication or demonstration. Robotic surgical console application has explored increased console allocation during procedures to allow the mentor and trainee to share instrument control, potentially leading to more efficient transfer of robotic skills. Limitations include dual console availability is needed and it necessitates the mentor be always physically present. Tele-mentoring is a means to overcome these limitations and improve training and application of surgeon skills in all scenarios. Our study demonstrates a safe and controlled environment to broaden the use of mentoring for the skilled or under-skilled surgeon delivering a continuum of improved robotic surgical application and training that can’t be limited by distance. Leveraging the development of technology in this manner can and should reduce medical errors leading to greater confidence in robotic surgical use.

Robotic surgery has been approved for a broad range of procedures including thoracic, gynecology, urology and general surgery. Robotic surgery continues to be integrated into new surgical procedures with greater utilization of the da Vinci Surgical system. We choose to utilize a broad approach and include many different types of cases in different specialties that utilize the da Vinci system when providing tele-mentoring. We demonstrated that tele-mentoring can be applied broadly for utilization across diverse robotic-assisted surgical procedures to help with surgical competence, teaching, and review. Tele-mentoring has the potential to extend the capabilities of surgeons with a more general skill set and efficiently share exceptionally specialized surgical expertise and other studies have concluded it as an effective training tool [11].

Adapting the da Vinci Xi Surgical system for connectivity beyond its fiber optic hard wire connections between consoles in the operating room is not a new and novel concept. Hinata et al. [29] described a robot tele-mentoring system created which used a dedicated optical fiber link based network with audio-visual transmission, three-dimensional monitors, and the TilePro function of the da Vinci robot, while Shin et al. [11] used Connect™ to remotely connect via wireless and wired internet connections for tele-mentoring. Our application on the other hand involved using the simple connection of Ethernet within the closed military network allowing secure and dedicated lines of connectivity. There was no latency or connectivity issues and security of information was not compromised. Our study did include enabling hard-ware components to provide tele-mentoring. Interestingly, the VTC network is integrated throughout all DoD facilities. This infrastructure allows surgeons to connect to surgical procedures from any military facility across the world. The interconnectivity of this system supports tele-mentoring across military institutions from military treatment facilities and, in the future, to forward austere environments.

There were limitations to the present study. Due to the logistical complexity of the study, we enrolled a relatively small number of cases. Also our study did not have a control group and was not randomized. Further limitations included only having one mentor to review the cases rather than having multiple mentors and collecting responses from most but not all individuals involved in the study. Additionally, our study did not test the effectiveness of mentoring by having less trained personnel proceed forward with surgical cases while supervised by a remote telementor. The local staff surgeons were highly trained and performing surgeries they commonly completed in their surgical practices. We also were constrained to only verbal mentor feedback with no visual feedback options through telestration. Lastly, Cisco Webex DX80 VTC system is a 2D display and wasn’t able to project the 3D imagery and telestration sent from the da Vinci Xi surgical system. Overall, these factors limit the ability to generalize our findings.

**Fig. 3:**
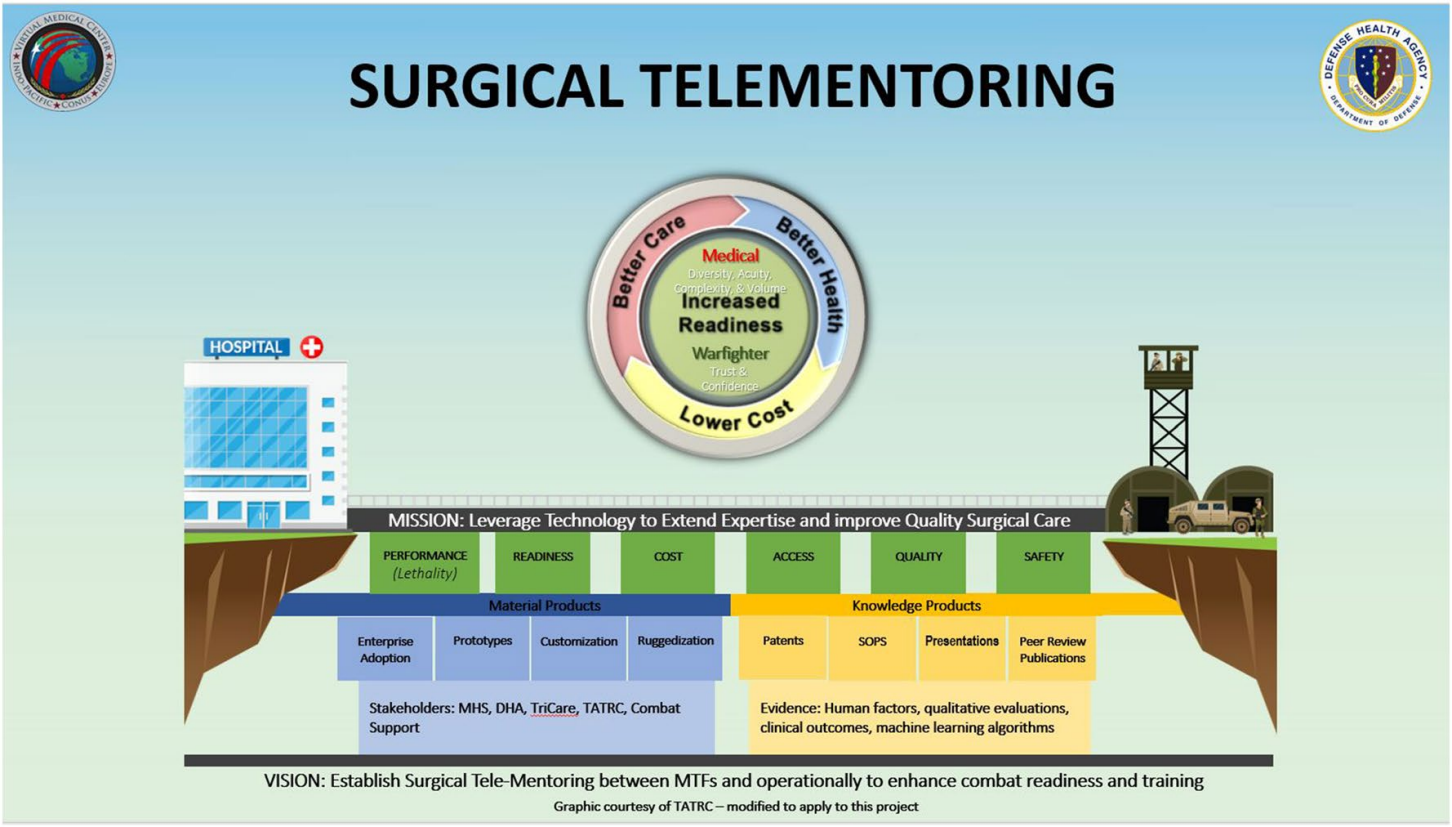
Vision—establish surgical tele-mentoring from military treatment facilities to support operational forces

Our novel interface of tele-mentoring on the Intuitive da Vinci Xi Surgical system is currently the only means to telementor on the most used robotic console in the world. This interface allows us to leverage the unique connective avenues and displays hardwired in to the military system. Further testing and adaptations will be necessary to make tele-mentoring on this platform a standard of care for training and robotic surgical cases. Future testing across broader distances will be necessary to prove the secure networks are without latency, connectivity, or security concerns. Attempts at 3D integration into Cisco displays and video teleconferencing should be explored, while also attempting to add further operating room data transfer to the mentor to limit ‘blind spots’ for tele-mentoring application. Tele-mentoring in itself needs defined metrics of performance and communication outlined to train mentors in the skills necessary for the most efficient enabled sustained deliberate practices on this platform. Continued application and use will help to identify these metrics and give a means of developing and training mentors to help create a pool of proficient best trained improving robotic surgeons across the military (Fig. 3). Future studies will further characterize the experience of both the surgical and mentoring teams in terms of evaluating the current technology, as well as the existing procedures that guide the establishment and maintenance of interpersonal/professional communication required for effective interaction between the two teams, and ultimately the best possible surgical outcome.

## Conclusion

Surgical tele-mentoring is safe and efficacious in providing remote surgical consultation to local surgeons utilizing robotic surgery technology in a military institution. Operative teams perceived this capability as beneficial and reliable audio-visual connectivity was demonstrated between the main operative room and the Virtual Medical Center. Reliable connectivity, lack of latency and security of information transferred were the conditions facilitated during this study eliminating these commonly experienced obstacles seen in previous studies. Tele-mentoring facilitated has the means to increase military readiness, access to care, increase quality of surgical expertise and improve patient safety for the service member. Further study is needed to develop surgical tele-mentoring capability to improve patient care without geographic limitations during times of peace, war, and pandemic outbreaks.

## Data Availability

Data is available upon request for the methods and materials related to this project.

## Declarations

### Awards

Second Place—General Surgery Category, award at SAGES Military Surgical Symposium 2022 Denver, CO.
